# Expectations of duplicate gene retention under the gene duplicability hypothesis

**DOI:** 10.1186/s12862-023-02174-2

**Published:** 2023-12-14

**Authors:** Amanda E. Wilson, David A. Liberles

**Affiliations:** Department of Biology and Center for Computational Genetics and Genomics, Temple University, 1900 N. 12th Street, Philadelphia, PA 19122 USA

**Keywords:** Gene duplication, Polyploidy, Probabilistic modeling, Molecular evolution, Comparative genomics, Mutational opportunity

## Abstract

**Background:**

Gene duplication is an important process in evolution. What causes some genes to be retained after duplication and others to be lost is a process not well understood. The most prevalent theory is the gene duplicability hypothesis, that something about the function and number of interacting partners (number of subunits of protein complex, etc.), determines whether copies have more opportunity to be retained for long evolutionary periods. Some genes are also more susceptible to dosage balance effects following WGD events, making them more likely to be retained for longer periods of time. One would expect these processes that affect the retention of duplicate copies to affect the conditional probability ratio after consecutive whole genome duplication events. The probability that a gene will be retained after a second whole genome duplication event (WGD2), given that it was retained after the first whole genome duplication event (WGD1) versus the probability a gene will be retained after WGD2, given it was lost after WGD1 defines the probability ratio that is calculated.

**Results:**

Since duplicate gene retention is a time heterogeneous process, the time between the events (t1) and the time since the most recent event (t2) are relevant factors in calculating the expectation for observation in any genome. Here, we use a survival analysis framework to predict the probability ratio for genomes with different values of t1 and t2 under the gene duplicability hypothesis, that some genes are more susceptible to selectable functional shifts, some more susceptible to dosage compensation, and others only drifting. We also predict the probability ratio with different values of t1 and t2 under the mutational opportunity hypothesis, that probability of retention for certain genes changes in subsequent events depending upon how they were previously retained. These models are nested such that the mutational opportunity model encompasses the gene duplicability model with shifting duplicability over time. Here we present a formalization of the gene duplicability and mutational opportunity hypotheses to characterize evolutionary dynamics and explanatory power in a recently developed statistical framework.

**Conclusions:**

This work presents expectations of the gene duplicability and mutational opportunity hypotheses over time under different sets of assumptions. This expectation will enable formal testing of processes leading to duplicate gene retention.

## Background

Gene duplication is an important process that gives rise to functional novelty in genomes through evolution [1–5]. Gene duplication can occur at a range of scales that are classified in two broad categories, whole genome duplication (WGD) and smaller scale duplication (SSD). While the nature of the duplicate gene fixation process is different between the events, as SSD events begin with a frequency of 1/2N in diploid species, while the initial frequency of a WGD event in a population of competing and breeding individuals is more complex and may be affected by things like hybridization and genomic instability [6].

Another major difference between the events is the nature of selection from dosage balance in the two events [7–9]. After WGD events, all genes are duplicated together with the interacting partners of their protein products [5, 10, 11]. After SSD events, genes are typically duplicated without the interacting partners of their protein products, leading to unfavorable stoichiometric imbalances [7, 8, 12]. The role of dosage balance in generating duplicate gene retention through eventual subfunctionalization has been modeled elsewhere [9, 13]. Because individual genes are affected differently by the processes of gene dosage balance, neofunctionalization and subfunctionalization, some genes are observed to be more inherently likely to be retained through gene duplication than other genes, which is referred to as the gene duplicability hypothesis [14–16].

Duplicability is seen as an inherent property of the genes related to their response to expression changes as well as the modularity and nature of their functions. The number of functions in a gene affects the ability to be retained through the subfunctionalization mechanism, while the number of mutationally achievable non-existing functions affects the ability to be retained through neofunctionalization [17–23]. These functions affect the retention probabilities and time dependent loss-rates as well [18]. In plants, lowly duplicable functions include genome stability maintenance and organelle-specific function (which needs to interact with organelle-encoded genes that may not be duplicated), while highly duplicable functions include signaling, transport, and metabolism [22]. Essential housekeeping genes (with core metabolic and informational functions) in Angiosperms that tend to be highly conserved across eukaryotes are often not retained as duplicates [23]. Young duplicate genes in plant genes were enriched in gene categories involved in stress responses, reflecting non-essential genes that play roles in specific environments [20]. Genes expressed in the nervous system have higher rates of retention in vertebrates [17]. Overall, genes within each functional category tended to have similar patterns of retention across paramecium species, with ribosomal proteins, transcription factors and intracellular signaling proteins being highly duplicable [21].

The number of interactions, network complexity, and dosage constraints also affect gene retention and duplicability, with large protein complexes less likely to be duplicated [16, 24–28]. Genes involved in the same pathway and protein complex share loss patterns, and genes with high expression in general tend to be retained at higher rates, while genes with a lot of interactions are less likely to be retained [21, 25, 29]. This might suggest both a role for gene number and for organismal effective population size in driving differential genome-specific retention patterns [13].

Different genes are typically seen as duplicable after SSD events and after WGD events, being biased towards certain functions [30–34]. In *Arabadopsis*, whole genome duplication events favor transcription factors, signal transducers, and organismal development, while these genes were not favored by small-scale duplication events, while genes involved in secondary metabolism and stress response tended to be favored by both large and small-scale duplication events [30]. Intrinsically disordered proteins are more duplicable after a whole-genome duplication compared to a small-scale duplication [31]. In humans, essential genes were more duplicable after a WGD event than after a small-scale event, while in yeast, they were more duplicable after small-scale events than after WGD events [32, 35, 36]. Because of the differences in their effects on the stoichiometric balance between interacting partners, WGD events lead to a slower initial duplicate gene loss rate while SSD favors fast initial loss rate [11, 12, 17, 20, 37–41]. In this study, we will focus on WGD events. Different genomes are likely to differ in the composition of their genome that includes genes that are commonly duplicable after WGD events as well as those that are contextually duplicable for that species.

Genes that have a small number of functions, are expressed in a small number of tissues and are highly sensitive to dosage shifts, such as heteromultimers would be examples of genes that fall into our dosage balance category (Dos). Examples of genes that are differentially subject to dosage balance processes without changing function to enable retention would be enzymes of glycolysis that differ in their multimerization status across the tree of life [42]. Additionally, the *Paramecium* genome is particularly sensitive to dosage constraints compared to other genomes post-WGD [41, 43].

Promiscuous genes that are expressed in multiple tissues at multiple developmental stages may have more opportunity to develop an alternative function (Alt_func, subfunctionalization or neofunctionalization). Examples of known neofunctionalized and subfunctionalized genes include diverged homologs in Atlantic salmon [44, 45]; 13% of homolog gene pairs in maize showed evidence of neofunctionalization [46]; 25% of homolog gene pairs in *Cyprinus carpio* largely sub- and neofunctionalized [39], while the rest of the gene pairs retained were through dosage or chance. Specific examples of the many genes that are known to have subfunctionalized are a yeast protein Orc1/Sir3 [47], transcription factor IIIA (gtf3a) and ovarian gtf3ab in teleost fish [48], and ruby2-ruby1 gene family in citrus [49]. Specific examples of genes known to have neofunctionalized are POLR3G and POLR3GL in mouse liver [50], and Retinoic Acid receptors in mammals, RARα, and RARγ [51]. Among angiosperm genes in the AP2/ERF gene family, which is involved in plant development and stress responses, those with broader expression patterns have higher rates of retention of duplicates than those with narrower expression profiles possibly suggesting they were retained through subfunctionalization [18]. Some genes have been found to neofunctionalize following subfunctionalization in a process coined subneofunctionalization, showing there isn’t always a hard distinction between subfunctionalization and neofunctionalization as seen in yeast and humans, and where subfunctionalization serves as a transition state to neofunctionalization [52, 53].

Other genes are unlikely to be retained through acquiring alternative functionalization. These genes are typically only retained for short periods of time following a duplication event due to drift (Non). Examples of genes that are typically found as only 1:1 orthologs across species are single copy genes across angiosperms, many of which are more conserved genes with essential housekeeping functions including those involved in photosynthesis, core metabolic processes, and the cell cycle [54, 55].

Different eukaryotic genomes have different fractions of such genes depending upon the environment that they live in, their effective population size, and other molecular, population level, and life history characteristics. For example, the paramecium genome is more dosage sensitive than other genomes post-WGD. Of retained homolog pairs, maize has 13% neofunctionalized [46], and *Cyprinus carpio* 25% sub- and neofunctionalized [39]. Genome content can very greatly, where for example the trypanosome genome [56] is structured very differently than the mammalian genome [57]. Trypanosome genomes seem to use duplication more than transcription factor binding evolution to modulate functional activity when compared with other genomes [58].

One naïve expectation of the gene duplicability hypothesis (GD) is that when consecutive whole genome duplication events have been observed in the lineage of a species, the genes that were retained after the first duplication event would be more likely to be retained after the second whole genome duplication event. This expectation was not found to hold in two genomes where this analysis was performed, in Atlantic salmon and in the orchid *Phalaenopsis equestris* [44, 59]. In the analysis of Atlantic salmon, specific gene properties associated with dosage and the gene duplicability hypothesis, like co-retention of interacting partners, were associated with preferential retention through consecutive events [44, 59, 60]. From seeing this incomplete picture of the gene duplicability hypothesis, it was clear that a more detailed modeling framework was needed to characterize gene duplicability and its expectations.

In addition to the gene duplicability hypothesis, we propose a hypothesis called the Mutational Opportunity (MO) hypothesis. We propose that the subfunctionalization and neofunctionalization processes gives rise to fewer future opportunities for subsequent subfunctionalization and neofunctionalization. This is structured as a nested model which encompasses the gene duplicability hypothesis, as opposed to a separate hypothesis. The mutational opportunity hypothesis is based on the fact that after a gene copy neofunctionalizes there are fewer novel mutations that are accessible to that gene, and after subfunctionalization there are fewer functions to specialize between the gene copies [61]. It should be noted that neofunctionalization has the potential to recharge subfunctionalization as a counter-balancing effect.

Most models for duplicate gene retention use a time-independent loss rate as a Poisson process which is known to be an inaccurate representation of duplicate gene retention probabilities as different processes have given rise to different time-dependent expectations [37, 62]. While more mechanistic Markov models of increasing levels of sophistication have been built [13, 63], the survival analysis framework of Konrad et al. 2011 [37] presents time-dependent gene loss probabilities associated with process-specific hazard functions that can be used to evaluate time-dependent expectations of the gene duplicability hypothesis. The survival analysis curves reflect averages of genes with certain characteristics in a genome. Differences in the average and variance of loss behavior can be modeled by changing the underlying parameters of the Konrad model. Here, we present this analysis framework together with the resulting dynamics under different sets of assumptions. The dynamics used differ from the naïve expectation above and can be used for explicit hypothesis testing about processes affecting duplicate gene retention in genomes. In this scenario, the parameters that are being explored for their effects on retention properties would be optimized with retention data using (for example) maximum likelihood inference. They could alternatively be estimated from the types of genes present in particular genomes. Either way, an explicit understanding of the expectations of the popular gene duplicability hypothesis are necessary to ultimately evaluate this idea.

Additionally, to model the expectations of the mutational opportunity model, we add an additional parameter indicating how frequently neo or subfunctionalized genes can no longer neo or subfunctionalize after the second whole genome duplication event. A mechanistic model for this process would have distributions for the numbers of subfunctionalizable functions and the potential number of unrealized mutationally accessible functions for a gene. Current understanding does not enable the construction of such models and the use of a category shifting parameter reflects a phenomenological approximation. The shifting parameter does not account for the increased hazard for genes with a reduced number of functions in the subfunctionalzation model but potentially averages this out with a shift of some genes to the nonfunctionalization model. Only a small number of sunfunctionalized genes with exactly 2 functions in the ancestor will be mechanistically described by this model. With this, models for gene duplicability and gene duplicability modulated by mutational opportunity are presented.

## Results and discussion

**Fig. 1:**
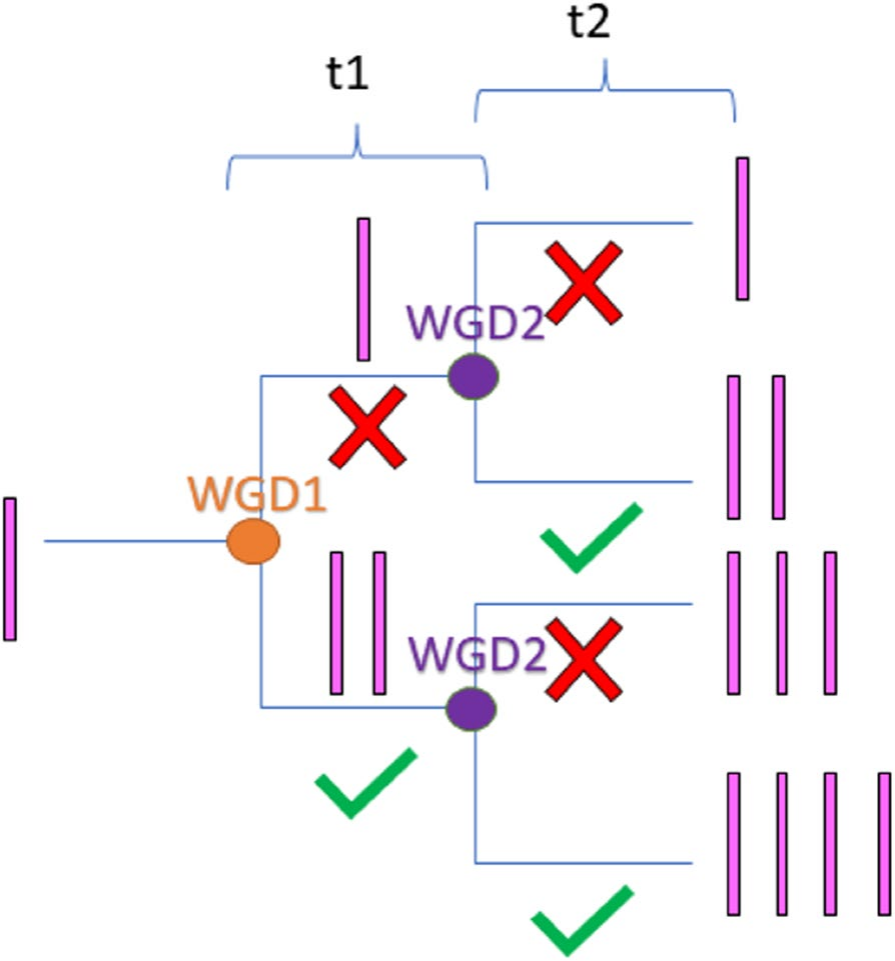
Decision tree for duplicate gene copies (pink) to be retained (green check) or lost (red x) during time t1 after WGD1 (orange) and or t2 after WGD2 (purple)

An experimental design has been created together with an associated probability ratio statistic to characterize patterns of duplicate gene retention in genomes with consecutive whole genome duplication events. The full dynamic behavior of gene retention under the gene duplicability hypothesis for consecutive whole genome duplication events (WGD1 and WGD2) with different time values for between the two duplication events (t1) and the time that has elapsed since the most recent duplication event (t2) will be examined in this work (Fig. 1). We used the survival analysis framework laid out in Konrad et al. 2011 [37] for the different behaviors of duplicate gene copy survival (Eq. 2) under different retention mechanisms(subfunctionalization, neofunctionalization, dosage balance, drift). In different genomes with different gene contents and multimerization patterns, we would expect these processes to play out with different proportions.

**Fig. 2:**
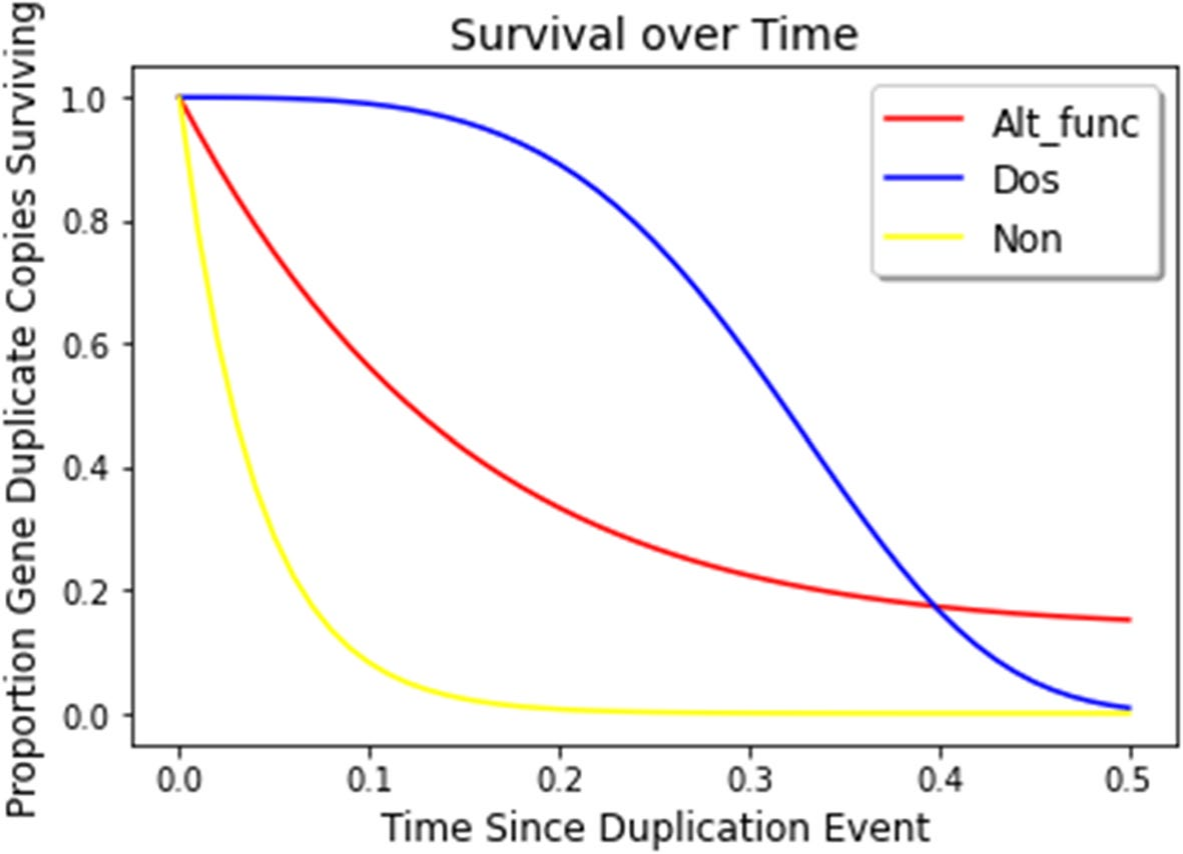
Survival curves of duplicate gene copies from wgd1 during t1 for the Alt_func category of genes (red line), the Dos category of genes (blue line), and the Non category of genes (yellow line)

We therefore explored genome evolution with different percentages of the starting genomes following each pattern of survival under each mechanism of retention. While some of the percentages are extreme, the systematic exploration is meant to understand the behavior of the system across the full range of parameters. Although the Neofunctionalization and Subfunctionalization category of genes from the Konrad et al. 2011 [37] paper have slightly different behavior to their instantaneous rate of gene duplicate copy loss over time, they are similar enough in their behavior that they are hard to differentiate from one another [63]. We chose to combine them into one category called the Altered-function category (Alt_func). The similarities in their displayed behavior of the instantaneous rate of gene duplicate copy loss includes that they are both concave up and decrease over time, meaning the rate at which duplicate loss occurs slows and levels off. Because of this, the survival curves are not readily distinguishable from each other as described in the Konrad et al. 2011 [37] paper; therefore our combined (Alt_func) category retains these characteristics and has a survival curve that is concave up and decreasing, and has an asymptote at a value above zero, meaning the rate of duplicate loss slows as there are fewer duplicates left, but some portion of duplicates are retained more permanently (Fig. 2). Both of the two processes also result in terminal retention, having the same effect on the test statistic. The dosage balance (Dos) category of genes displays the behavior where the instantaneous rate of loss of gene copy duplicates increases as the duplication events age, and therefore the survival curve is concave down and decreasing, meaning gene duplicates get lost faster, until it hits an inflection point where the rate of gene loss slows and has an asymptote at zero (Fig. 2). The category where retention is purely by chance, and the genes can only nonfunctionalize (Non), has a time-homogeneous instantaneous rate of loss of gene duplicate copies. For this category, all of the gene copies will eventually reach the point where they nonfunctionalize with enough time following the duplication event, so its survival curve of gene duplicate copies has an asymptote at zero as in the Dos category. Here the survival curve is also concave up and decreasing, like the Alt_func category, and has a gene loss rate that slows; however it decays much faster for reasons that are increasingly well understood [13] (Fig. 2). We calculated the probability ratio (p_ratio_) for consecutive whole genome duplication events (Eq. 1). Incorporating these different percentages of different categories, using the α parameter that represents the proportion of the initial genome that fall into each category, as shown in Eq. 3. For small values of t1, the duplicate genes may not resolve into a terminal fate (Alt_functionalize or Nonfunctionalize) before another round of whole genome duplication. This would result in four entirely redundant copies. In this case, the model for retention during t2 is identical to the model as if they had resolved, but also includes a possibility of losing three of the four redundant copies. The probability of such an event can be explicitly calculated but has not been included here. The current model assumes all genes are simply duplicated. However, it is important to have a model that is applicable for small values of t1 because real fish and plant genomes can have small t1 values.

**Fig. 3:**
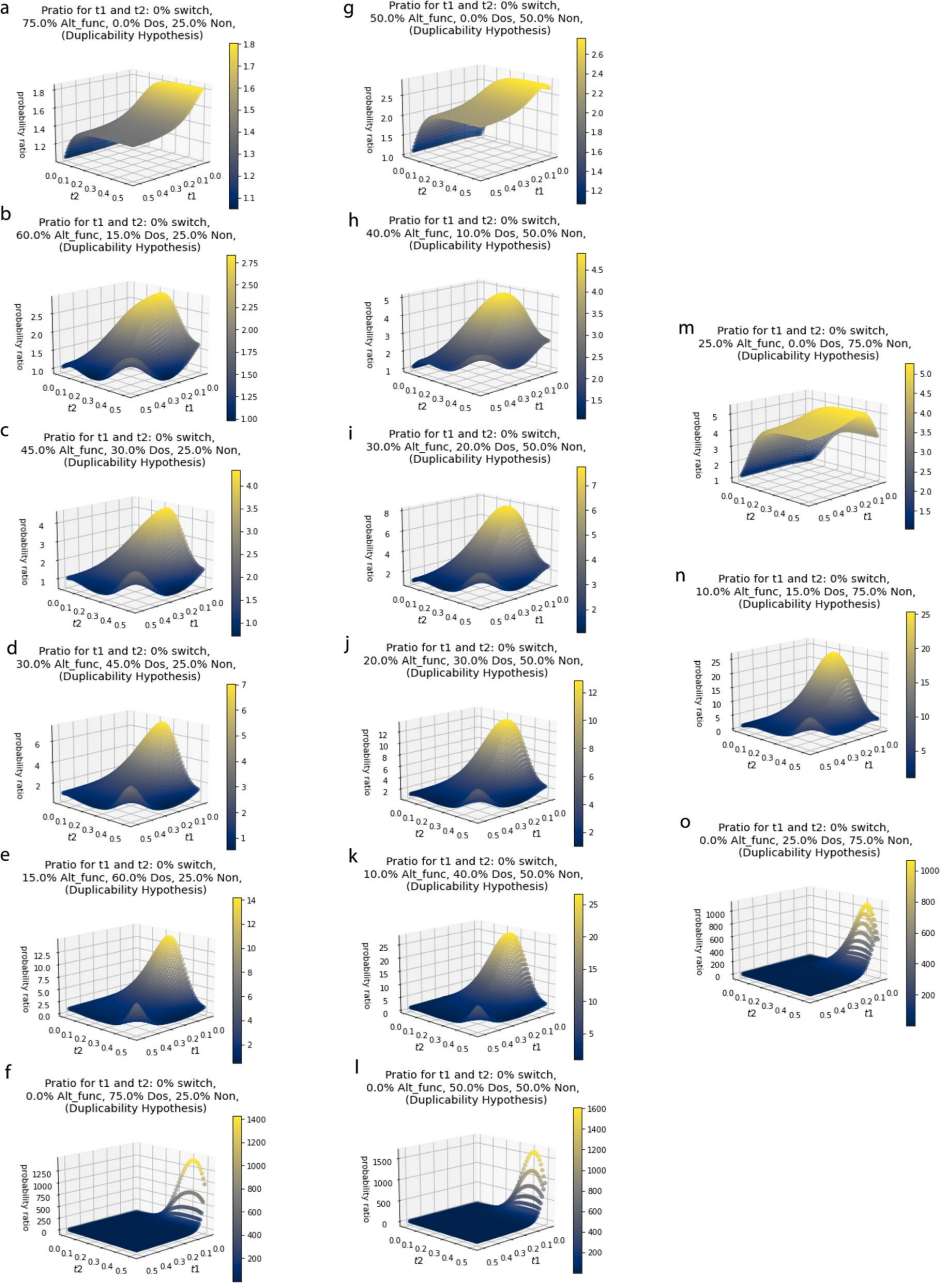
Gene duplicability – surface of the probability ratio over various times for t1 and t2 for given proportion of the starting genome in the Alt_func category, Dos category, and Non category (α_Alt_func_, α_Dos_, α_Non_ respectively).         **a** α_Alt_func_ = 0.75, α_Dos_ = 0.0, α_Non_ = 0.25, **b** α_Alt_func_ = 0.60, α_Dos_ = 0.15, α_Non_ = 0.25.         **c** α_Alt_func_ = 0.45, α_Dos_ = 0.3, α_Non_ = 0.25.         **d** α_Alt_func_ = 0.3, α_Dos_ = 0.45, α_Non_ = 0.25.         **e** α_Alt_func_ = 0.15, α_Dos_ = 0.6, α_Non_ = 0.25.         **f** α_Alt_func_ = 0.0, α_Dos_ = 0. 75, α_Non_ = 0.25.         **g** α_Alt_func_ = 0.5, α_Dos_ = 0.0, α_Non_ = 0.5.         **h** α_Alt_func_ = 0.4, α_Dos_ = 0.1, α_Non_ = 0.5.         **i** α_Alt_func_ = 0.3, α_Dos_ = 0.2, α_Non_ = 0.5.         **j** α_Alt_func_ = 0.2, α_Dos_ = 0.3, α_Non_ = 0.5.         **k** α_Alt_func_ = 0.1, α_Dos_ = 0.4, α_Non_ = 0.5.         **l** α_Alt_func_ = 0.0, α_Dos_ = 0.5, α_Non_ = 0.5.         **m** α_Alt_func_ = 0.25, α_Dos_ = 0.0, α_Non_ = 0.75.         **n** α_Alt_func_ = 0.1, α_Dos_ = 0.15, α_Non_ = 0.75.         **o** α_Alt_func_ = 0.0, α_Dos_ = 0.25, α_Non_ = 0.75

Figure 3 shows that the probability ratio depends on the t1 and t2 values. A p_ratio_ of 1 reflects that the genes are “unsorted”, meaning the probability of a gene being retained after a second event is the same regardless of its probability of being retained after the first event. A high p_ratio_ reflects genes are “sorted” such that the probability of being retained after the second event is very likely if it was retained in the first event, and very unlikely to be retained if it was not retained in the first event. We chose not to include the p_ratio_ for a t1 and t2 value of zero because it is undefined in our model, and we can reasonably assume that the two events cannot happen simultaneously and happened with enough time that the events have fixed before the moment of acquiring the data. The p_ratio_ values are highest for very short t1 values and slowly get smaller as t1 values get larger. A very high p_ratio_ for very small t1 values reflects that almost all genes that are likely to be retained in t2 are probably retained immediately following the second whole genome duplication event. This effect is seen for genes in the Dos and Alt_func categories. The p_ratio_ starts at 1 for small t2 values because the gene retention pattern is unsorted, and the p_ratio_ gradually gets higher for older t2 values, the speed at which it gets bigger varies depending on the proportions of the starting genome in each category. While the magnitude of p_ratio_ peaks depend on the proportions of the starting genome in each category, there is consistently the highest peak at short t1 values and medium to long t2 values. There is a secondary smaller peak at very long t1 and t2 values.

Figure 3 shows the probability ratio over a range of t1 and t2 values depend on the starting proportion of the genome in the Alt_func category, Dos category, and Non category (α_Alt_func_, α_Dos_, α_Non_ respectively). Three percentages from the Non category were used, 25% (Fig. 3a-f), 50% (Fig. 3g-l) and 75% (Fig. 3m-o). The remaining percent was then split between the Alt_func and Dos categories, with decreasing Alt_func and increasing Dos by 10–15%, including 0% of each. Figure 3 shows that with increasing Dos and decreasing Alt_func, the peak probability ratio reached is higher, particularly in those with a short to moderate t1 and a moderate to long t2, and those with long t1 and t2 values, with a greater distribution of probability ratios that can be achieved. These very high peaks are driven by high dosage because it leads to the retention of more of the genes for longer in both t1 and t2, creating a more sorted effect. For those with none in the Dos category, the peak has the appearance of a raised plateau, and never peaks as high as with genomes containing genes in the Dos category. Of the graphs that have no Dos genes (Fig. 3a, g and m), as the percentage of initial genes in the Non increases and Alt_func decreases, the higher the probability ratio plateau peaks, particularly for younger t1 events.

Importantly to note, we surprisingly see some p_ratio_ values below one, even for those under the gene duplicability hypothesis. This happens for those with relatively large percentage in the Dos category for shorter t1 values but long t2 values. This is because at shorter time values, those in the Dos category are significantly more likely to be retained than at long time values, so it leads to “sorting” in the opposite direction, where those that were retained at short time values in t1 are more likely to be lost in the long t2 value. A similar, but opposite pattern is observed short t2 values and long t1 values. These effects occur because Dos genes are retained over shorter time periods but are ultimately lost after long periods while Alt-func genes that are lost are lost over shorter time periods but ultimately retained over long periods with a reduced loss rate.

**Fig. 4:**
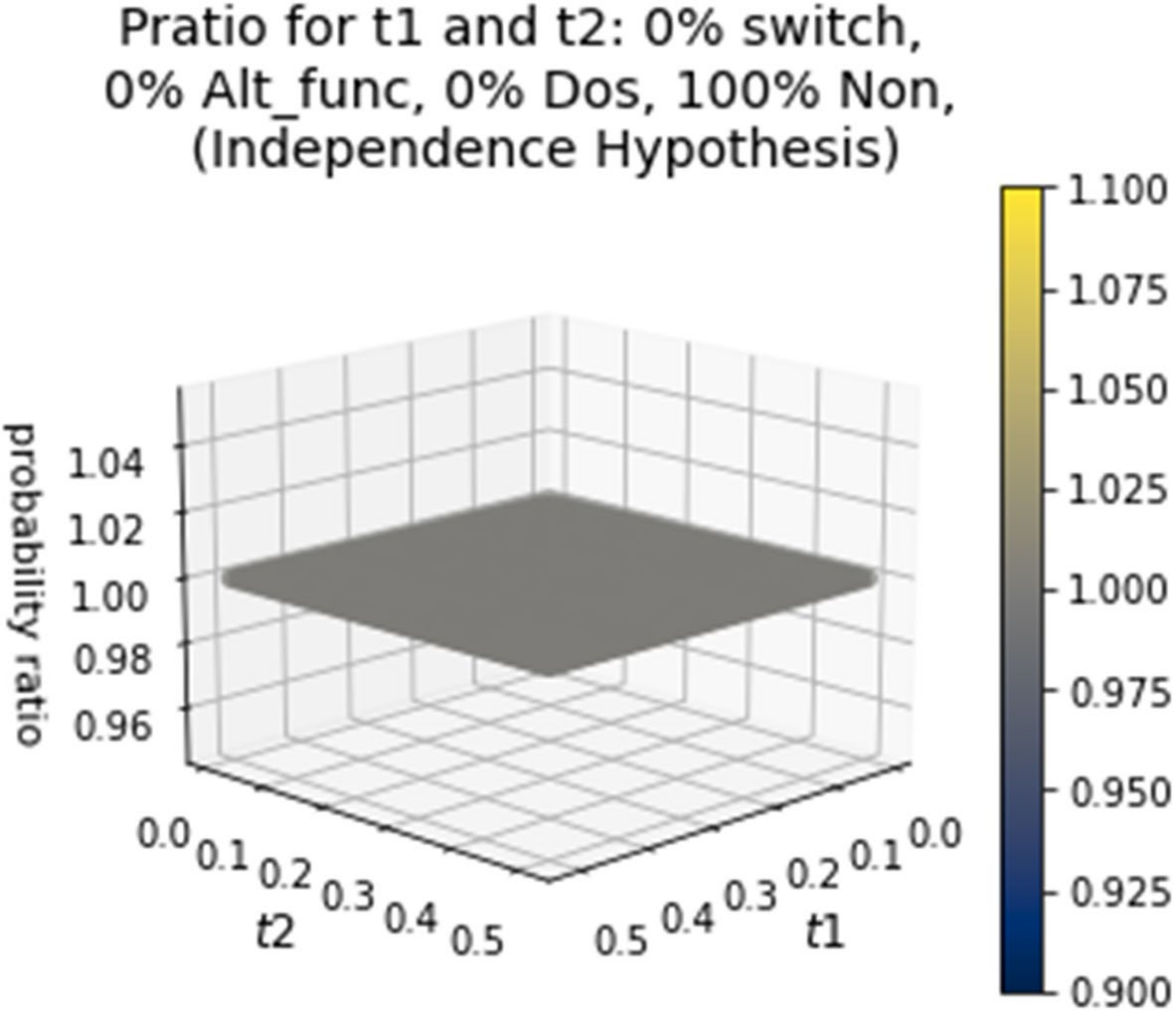
Independence – probability ratio over various times for t1 and t2 if 100% of the genes in the genome had the same probability of being retained. This example has all the genes being in the Non category. This is a model of the independence hypothesis, the null hypothesis to the gene duplicability hypothesis. It is presented as a confirmation of expectations

Figure 4 models the independence hypothesis and shows the probability ratio over a range of t1 and t2 values if 100% of the genes in the genome had the same probability of being retained. This example has all the genes being in the Non category. The figure confirms expectation, that the probability ratio is equal to one regardless of how old either duplication event is. These findings show that the gene duplicability hypothesis expectations are distinct from the expectations under the independence hypothesis over the range of t1 and t2 values; however, the gene duplicability hypothesis and mutational opportunity hypothesis have t1 and t2 values that can lead to a p_ratio_ of 1.

**Fig. 5:**
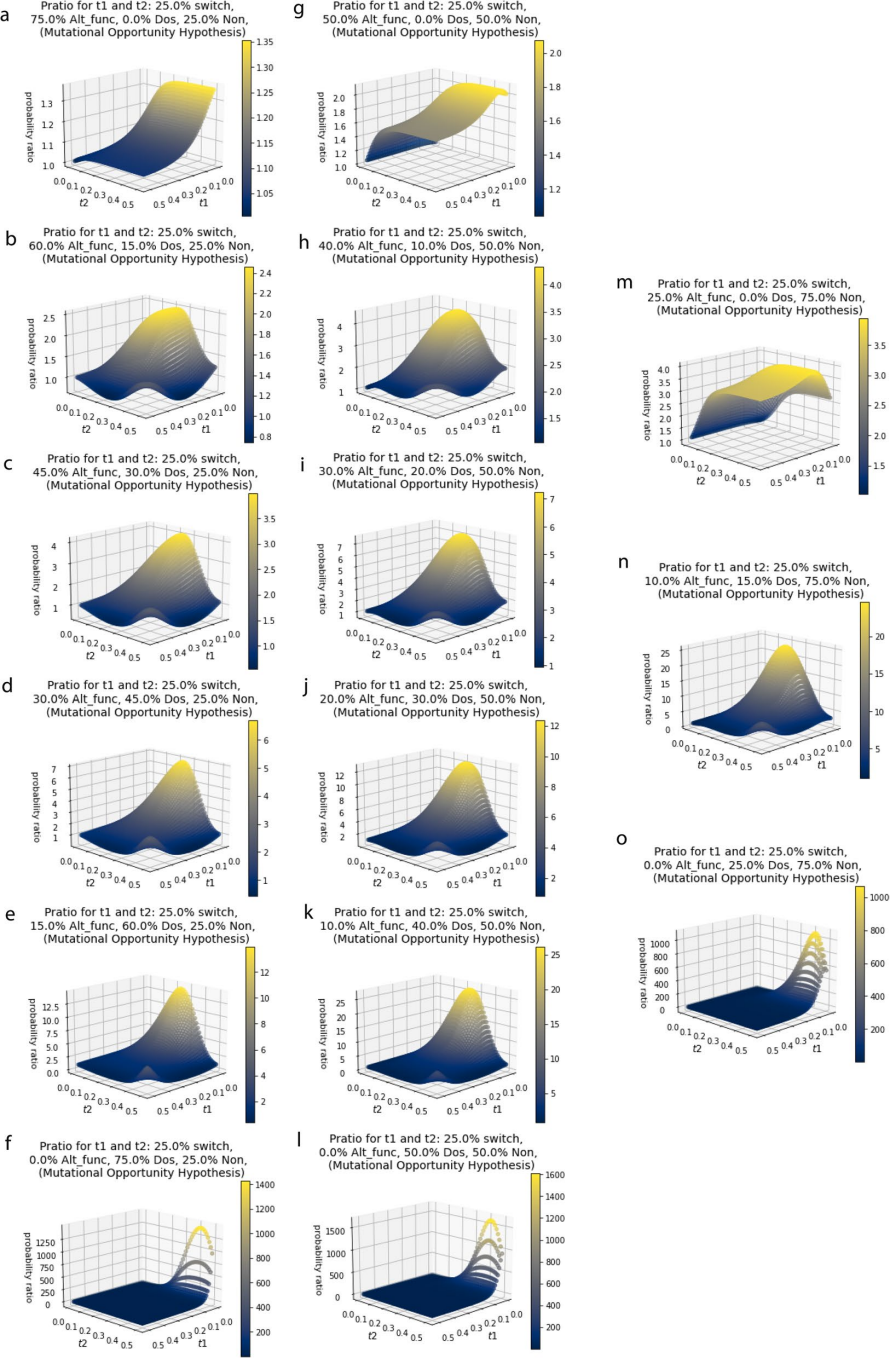
Mutational opportunity – surface of the probability ratio over various times for t1 and t2 for given proportion of the starting genome in the Alt_func category, Dos category, and Non category (α_Alt_func_, α_Dos_, α_Non_ respectively) and β_switch_mo_ equal to 0.25, which is the proportion of the Alt_func category that switches to the Non category during t2 because they cannot neofunctionalize or subfunctionalize again. **a** α_Alt_func_ = 0.75, α_Dos_ = 0.0, α_Non_ = 0.25, **b** α_Alt_func_ = 0.60, α_Dos_ = 0.15, α_Non_ = 0.25.         **c** α_Alt_func_ = 0.45, α_Dos_ = 0.3, α_Non_ = 0.25.         **d** α_Alt_func_ = 0.3, α_Dos_ = 0.45, α_Non_ = 0.25.         **e** α_Alt_func_ = 0.15, α_Dos_ = 0.6, α_Non_ = 0.25.         **f** α_Alt_func_ = 0.0, α_Dos_ = 0. 75, α_Non_ = 0.25.         **g** α_Alt_func_ = 0.5, α_Dos_ = 0.0, α_Non_ = 0.5.         **h** α_Alt_func_ = 0.4, α_Dos_ = 0.1, α_Non_ = 0.5.         **i** α_Alt_func_ = 0.3, α_Dos_ = 0.2, α_Non_ = 0.5.         **j** α_Alt_func_ = 0.2, α_Dos_ = 0.3, α_Non_ = 0.5.         **k** α_Alt_func_ = 0.1, α_Dos_ = 0.4, α_Non_ = 0.5.         **l** α_Alt_func_ = 0.0, α_Dos_ = 0.5, α_Non_ = 0.5.         **m** α_Alt_func_ = 0.25, α_Dos_ = 0.0, α_Non_ = 0.75.         **n** α_Alt_func_ = 0.1, α_Dos_ = 0.15, α_Non_ = 0.75.         **o** α_Alt_func_ = 0.0, α_Dos_ = 0.25, α_Non_ = 0.75

**Fig. 6:**
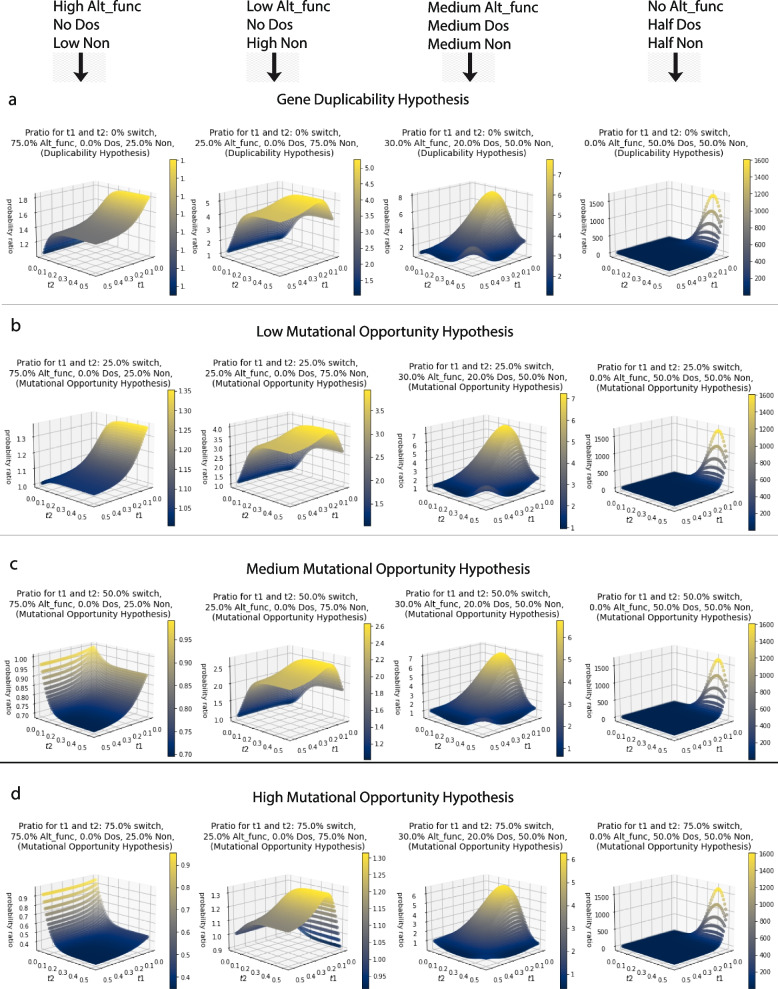
Comparison of the expected p_ratio_ values under the **a** gene duplicability hypothesis and **b**-**d** mutational opportunity hypothesis with different values of β_switch_mo_ (0.25, 0.50, 0.75 respectively), which is the proportion of the Alt_func category that switches to the Non category during t2 because they cannot neofunctionalize or subfunctionalize again. These were shown for High Alt_func, No Dos, and Low Non (75% Alt_func, 0% Dos, 25% Non), Low Alt_func, No Dos, and High Non (25% Alt_func, 0% Dos, 75% Non), Medium Alt_func, Dos and Non (30% Alt_func, 20% Dos, 50% Non), and No Alt_func, Half Dos and Half Non (0% Alt_func, 50% Dos, 50% Non). This shows that genomes with genes in the Alt_func category have smaller p_ratio_s for those that are more likely to lose their ability to be retained again in t2. Genomes without genes in the Alt_func do not change between the gene duplicability hypothesis and the mutational opportunity hypothesis. Genomes with extremely large Alt_func categories and are extremely unlikely to be retained in t2 under this mechanism have p_ratio_s exclusively less than one, and with a surface topology that changes the most from the gene duplicability hypothesis

In addition to the treatment of duplicates that have not fully resolved in the Alt_func category after a short t1 period, the explicit gene duplicability model assumes that the retention probabilities do not change in a second event following retention in the first event. Subfunctionalization reduces the probability of further subfunctionalization because there are fewer functions to subfunctionalize in the second round. The same is true for neofunctionalization reducing the probability of future neofunctionalization. However, it is also true that neofunctionalization can enable future retention by subfunctionalization. We describe this model, with these added layers of complexity, as a distinct hypothesis we call, mutational opportunity. Mutational opportunity was modeled by the introduction of a parameter that shifts the category for some of the retained pairs in the Alt_func category for the second retention period (Eq. 4). Like in the gene duplicability model, it becomes more complicated if accounting for duplicates that have not fully resolved after t1. We see a similar pattern for those under the mutational opportunity hypothesis as we do for those in the gene duplicability hypothesis, but the 3D surface is suppressed with lower p_ratio_ values, especially at long values of t1 and t2 (Fig. 5). There is a bigger suppression effect for those with more in the Alt_func category, and for those that have more of the Alt_func category switching to the nonfunctionalization category in the second WGD event (Fig. 6). For those without genes in the Alt_func category we do not see any effect, which is to be expected (Figs. 5 and 6). We only see a topological change when Alt_func is very large and the percentage that switch to nonfunctionalization in t2 (β_switch_mo_) is also exceptionally large, especially when there are no genes in the Dos category (Fig. 6). For example, the genome with 75% in the Alt_func category and 25% in the Non category, and a β_switch_mo_ of 75%, the p_ratio_ actually peaks close to one for short t2 and the p_ratio_ gets smaller as t2 gets larger, pretty much regardless of t1 values, however these values are likely outside the realm of realism (Fig. 6).

## Conclusions

We characterized the behavior of gene duplicate copy survival for genomes that experienced consecutive whole genome duplication events under the gene duplicability hypothesis mutational opportunity hypotheses. We modeled the gene duplicability hypothesis as the starting genome having some proportion of genes that are dosage sensitive and some having opportunity for neofunctionalization or subfunctionalization. We modeled the mutational opportunity hypothesis as also incorporating a proportion of genes that neofunctionalized or subfunctionalized as not being able to subsequently neo or subfunctionalize again. The model predicted expected probability ratios for survival of gene copies over different t1 and t2 values and predicts that the probability ratio (p_ratio_) expected will depend on the proportion of the starting genome that are sensitive to dosage or having opportunity for neo- or subfunctionalization. In addition, the expected p_ratio_ depends on the t1 and t2 values. Our finding shows that the gene duplicability hypothesis predicts distinct expectations for p_ratios_ from the independence hypothesis. The independence hypothesis predicts a p_ratio_ of one regardless of t1 and t2 values or the proportion of the genome sensitive to dosage pressure and with opportunity to neo- or subfunctionalize, while the gene duplicability hypothesis predicts the p_ratio_ to be different for different t1 and t2 values and depending on the proportion of starting genes in each category. It is unclear if the gene duplicability hypothesis is distinguishable from the mutational opportunity hypothesis, except under extreme circumstances, but does result in lower p_ratio_ values than what would be expected under the gene duplicability hypothesis.

The p_ratio_ of less than one is possible under the gene duplicability models, although most are greater than 1 for those that include a terminal retention process. Some time-point combinations give rise to an expectation of or close to 1. This can happen for long t2 values and will depend upon if the hazard rate of the Alt_func model decays to zero or a value above zero. Interestingly, different sets of time points give rise to expectations of a ratio greater than 1 when there is no terminal retention process available to any of the genes. Overall, the set of genes subject to dosage balance processes (for example those that obligately form heteromultimers) leads to a very large expected p_ratio_ with short t1 values, which decay with increasing t2 as described. The pattern described is the same under the mutational opportunity model, although there are smaller expected p_ratios_ across the board.

The surface is flatter and closer to one for genomes with a smaller proportion of starting genes under dosage balance forces. Genomes with a lower proportion of genes with opportunity for neofunctionalization or subfunctionalization and higher proportion of genes under dosage balance effects and can only be retained by chance have a steeper surface with higher peaks, particularly for short to moderate values of t1 and long t2, however long t1 and t2 values also have a small peak. However, the gene duplicability hypothesis does give rise to a p_ratio_ of one and less than one for certain t1 and t2 values and different proportions of starting genes in each category. More work is needed to identify more p_ratio_ data points from additional genomes with different t1 and t2 values to determine which hypothesis best explains observed data in genomes to illuminate evolutionary mechanisms.

In applying models to genomic data, it is assumed that the clades being examined will have similar starting fractions of genes in each category, which becomes a set of parameters to estimate using likelihood-based methods. This is a reasonable assumption for comparing species that are related and have broadly similar lifestyles, such as monocot species together or teleost fish together.

## Methods

To explore patterns of duplicate gene retention, we examined consecutive whole genome duplication events and the probability of both gene duplicate copies being retained after a second duplication event conditional on whether they were both retained after the first duplication event, using a summary statistic called the probability ratio (p_ratio_) given by Eq. 1. The first duplication event we refer to as WGD1 and the second WGD. The time between the two events is t1 and the time since the most recent event is t2.

1 $$pratio=\frac{probability\;of\;survival\;in\;t2\;\vert\;survived\;in\;t1}{probability\;of\;survival\;in\;t2\;\vert\;lost\;in\;t1}=\frac{2\ast S\left(t_1\right)\ast S\left(t_2\right)}{(1-S\left(t_1\right))\ast S\left(t_2\right)}$$

2 $${s\left(t\right)=e}^{(-dt-f\sum _{n=0}^{\infty }\frac{{\left(-b\right)}^{n}*{t}^{c*n+1}}{c*n\left(n!\right)+n!})}$$

3 $$\begin{array}{c}{pratio}_{gene\;duplicability}=\\\frac{{\left(P\left(survival\;in\;t2\;\right|survived\;in\;t1\right)}_{Alt_{func}}\ast\%_{Alt_{func}})+{\left(P\left(survival\;in\;t2\;\right|survived\;in\;t1\right)}_{Dos}\ast\%_{Dos})+{\left(P\left(survival\;in\;t2\;\right|survived\;in\;t1\right)}_{Non}\ast\%_{Non})}{{\left(P\left(survival\;in\;t2\;\right|lost\;in\;t1\right)}_{Alt_{func}}\ast\%_{Alt_{func}})+{\left(P\left(survival\;in\;t2\;\right|lost\;in\;t1\right)}_{Dos}\ast\%_{Dos})+{\left(P\left(survival\;in\;t2\;\right|lost\;in\;t1\right)}_{Non}\ast\%_{Non})}\\=\frac{2\ast\alpha_{Alt\_func}\ast S_{Alt\_func}\left(t_1\right)\ast S_{Alt\_func}\left(t_2\right)+2\ast\alpha_{Dos}\ast S_{Dos}\left(t_1\right)\ast S_{Dos}\left(t_2\right)+2\ast\alpha_{Non}\ast S_{Non}\left(t_1\right)\ast S_{Non}\left(t_2\right)}{\left(1-S_{Alt\_func}\left(t_1\right)\right)\ast\alpha_{Alt\_func}\ast S_{Alt\_func}\left(t_2\right)+\left(1-S_{Dos}\left(t_1\right)\right)\ast\alpha_{Dos}\ast S_{Dos}\left(t_2\right)+\left(1-S_{Non}\left(t_1\right)\right)\ast\alpha_{Non}\ast S_{Non}\left(t_2\right)}\ast\frac{\left(1-S_{Alt\_func}\left(t_1\right)\right)\ast\alpha_{Alt\_func}+\left(1-S_{Dos}\left(t_1\right)\right)\ast\alpha_{Dos}+\left(1-S_{Non}\left(t_1\right)\right)\ast\alpha_{Non}}{2\ast\alpha_{Alt\_func}\ast S_{Alt\_func}\left(t_1\right)+2\ast\alpha_{Dos}\ast S_{Dos}\left(t_1\right)+2\ast\alpha_{Non}\ast S_{Non}\left(t_1\right)}\end{array}$$

4


The process of gene duplicate copy retention is time heterogeneous and differs depending on the mutation and selective forces on each specific gene. To model this, we used the survival analysis framework designed by Konrad et al. 2011 [37], that describes process-specific hazard functions of duplicate gene copies (Eq. 2). To model the process-specific time-heterogeneity of duplicate copy retention, they gave different parameter values to use for categories of genes with different mutation and selective forces that affect the processes of retention available to them. The parameters used were b, c, d, and f. Parameters f + d represent the rate at which fully redundant genes get lost from the genome, and this diminishes to d, which is the rate at which non-duplicated genes are lost. Parameters b and c describe the shape of the curve, i.e. the dynamics/behavior of the process when moving from the instantaneous rate to the asymptotic rate. These parameters are not explicit values that can be experimentally determined, but they are summaries of the underlying biological processes, and can be determined through model fitting to existing data. The four categories of genes described in the Konrad et al. 2011 [37] model were (1) those that are sensitive to stochiometric balance effects that would lead to selective pressure for both copies to be retained by dosage balance/compensation or one of the copies lost through nonfunctionalization (Dos), (2) those that cannot be retained through any given process, and therefore both can only be retained by chance that one has yet to nonfunctionalize (Non), (3) those that have the potential for both copies to be retained through subfunctionalization or one of the copies lost through nonfunctionalization, and (4) those that have the potential for both copies to be retained through neofunctionalization of one of the copies or one of the copies lost through nonfunctionalization. The survival curves are not easily differentiable between gene copy pairs retained through the process of subfunctionalization and the neofunctionalization process, so we combined these retention mechanisms in our model (Alt_func) [38].

We incorporated the survival analysis framework into our probability ratio statistic (Eq. 1). Then, using this framework, we could test the gene duplicability hypothesis (Eq. 3), where we assume that some percentage of the genome has genes that fit into one of the three categories. Both gene copies can be retained through some mechanism of retention, Alt_func and Dos, or they can both be retained only by chance that one of them has yet to have nonfunctionalized, Non. This models the gene duplicability hypothesis because it assumes that genes have some inherent predisposition to be retained through specific mechanisms, either through their function (for example: GO terms) or number of interacting partners. The α parameter represents the proportion of the initial genome that fall into each category. Each surviving proportion of duplicate gene copies in t1 (s(t1)) is in Eqs. 1 and 3 are multiplied by 2 because there will be two of those copies that can be duplicated in the second wgd event. The probability of those lost in t1 are 1– the surviving proportion of duplicate gene copies (1-s(t1)).

Using a nested framework, we added an additional parameter for the mutational opportunity hypothesis that represents the portion of genes that have subfunctionalized and/or neofunctionalized lose the ability to subfunctionalize or neofunctionalize again after the second duplication event (Eq. 4). The percentage of retained genes that “switch” from this alternative-functionalization category in the second duplication event is represented by the beta value (β_switch_mo_). Therefore, the probability of surviving duplicate gene copies in the alt_func category is split into two parts, the first part being the percentage of genes that can be retained again, and the second part being the percentage of genes that cannot be retained again, therefore they switch to the nonfunctionalization (non) category for t2.

We designed a python script, which can be found at https://github.com/aewilson96/Gene_Duplicability_Models, to calculate the p_ratio_ for the gene duplicability hypothesis and mutational opportunity hypothesis (Eqs. 3 and 4), using this survival analysis framework. The parameters used to generate the figures provided were chosen from the Konrad et al. 2011 [37] paper, and adjusted for visual clarity. These included n_max = 100, b_Alt_func = 10.0, c_Alt_func = 2.37, d_Alt_func = 0.00054, f_Alt_func = 5.84, b_Dos = -17.0, c_Dos = 0.2573, d_Dos = -0.000028, f_Dos = 0.000028, b_Non = 0, c_Non = 1, d_Non = 20, and f_Non = 5. The script calculates the p ratio for genomes with different starting proportions in each category (Alt_func, Dos, or Non) for 50 time points from t = 0.0 to t = 0.5 for both t1 and t2, so it includes every combination of these time points to the hundredths. In Fig. 4, we showed the independence hypothesis, so we assumed 100% of the genome was acting under the same model. For Fig. 3a-o, α_Alt_func_, α_Dos_, α_Non_ used were as shown in Table 1. For over fifty time points, 3D surface plots were made for each combination of α values. These combinations help provide a visual range of what the surface figures look like for the gene duplicability hypothesis and how it can be differentiated from the independence hypothesis. The same α_Alt_func_, α_Dos_, α_Non_ (as shown in Table 1) and fifty time points were used to generate 3D surface plots for the mutational opportunity hypothesis with a β_switch_mo_ value of 25% (Fig. 5a-o). Figure 6 shows a few select comparisons between the gene duplicability and mutational opportunity expectations with β_switch_mo_ value of 25%, 50% and 75%.

**Table 1:** Values of the α parameter which represents the proportion of the initial genome for each category, Alt_func, Dos and Non, where α_Alt_func_ + α_Dos_ + α_Non_ = 1.

	Α_Alt_func_	α_Dos_	α_Non_
Figures 3a and 5a	0.75	0.0	0.25
Figures 3b and 5b	0.60	0.15	0.25
Figures 3c and 5c	0.45	0.3	0.25
Figures 3d and 5d	0.3	0.45	0.25
Figures 3e and 5e	0.15	0.6	0.25
Figures 3f and 5f	0.0	0.75	0.25
Figures 3g and 5g	0.5	0.0	0.5
Figures 3h and 5h	0.4	0.10	0.5
Figures 3i and 5i	0.3	0.2	0.5
Figures 3j and 5j	0.2	0.3	0.5
Figures 3k and 5k	0.1	0.4	0.5
Figures 3l and 5l	0.0	0.5	0.5
Figures 3m and 5m	0.25	0.0	0.75
Figures 3n and 5n	0.1	0.15	0.75
Figures 3o and 5o	0.0	0.25	0.75

## Data Availability

All code to enable the work presented here is available at https://github.com/aewilson96/Gene_Duplicability_Models. No other data was used for this study.

## Abbreviations

Alt_Func: Is the altered function model for duplicate gene retention consistent with neofunctionalization or subfunctionalization
Dos: Is the dosage balance model for duplicate gene retention
Non: Is the nonfunctionalization model for duplicate genes
P_ratio_: Is the conditional probability ratio
SSD: Refers to smaller scale duplication
T1: Is the time between consecutive WGD events
T2: Is the time since the most recent WGD event
WGD: Refers to whole genome duplication
Mut Op or MO: Refers to the Mutational Opportunity Hypothesis
Gene Dos or GD: Refers to the Gene Duplicability Hypothesis
